# Case report: long-term survival of an infant syndromic patient affected by atypical teratoid-rhabdoid tumor

**DOI:** 10.1186/1471-2407-13-100

**Published:** 2013-03-05

**Authors:** Piergiorgio Modena, Iacopo Sardi, Monica Brenca, Laura Giunti, Anna Maria Buccoliero, Bianca Pollo, Veronica Biassoni, Lorenzo Genitori, Manila Antonelli, Roberta Maestro, Felice Giangaspero, Maura Massimino

**Affiliations:** 1 Unit of Experimental Oncology 1, Centro di Riferimento Oncologico, Aviano, 33081, Italy; 2 Department of Onco-hematology, Meyer Pediatric Hospital, Firenze, Italy; 3 Department of Pathology, C. Besta Neurologic Institute, Milano, Italy; 4 Department of Pediatric Oncology I.R.C.C.S, “Istituto Nazionale Tumori”, Milano, 20131, Italy; 5 Department of Neuro-Surgery, Meyer Pediatric Hospital, Firenze, Italy; 6 Department of Pathology, Sapienza University, Policlinico Umberto I, Roma, 00161, Italy; 7 I.R.C.C.S. “Neuromed”, Pozzilli, Italy

**Keywords:** Atypical teratoid rhabdoid tumor, ATRT, SMARCB1/INI1, Medulloblastoma, MLPA

## Abstract

**Background:**

Atypical teratoid rhabdoid tumor (ATRT) patients display a dismal median overall survival of less than 1 year. A consistent fraction of cases carries de-novo *SMARCB1/INI1* constitutional mutations in the setting of the “rhabdoid tumor predisposition syndrome” and the outcome is worst in infant syndromic ATRT patients.

**Case presentation:**

We here describe a patient affected by mosaic Klinefelter syndrome and by rhabdoid tumor predisposition syndrome caused by constitutional *SMARCB1/INI1* heterozygous mutation c.118C>T (Arg40X). Patient’s ATRT primary tumor occurred at 2 years of age concurrent with metastatic lesions. The patient was rendered without evidence of disease by combined surgery, high-dose poli-chemotherapy and craniospinal irradiation, followed by autologous hematopoietic stem cell transplantation. At the onset of a spinal lesion 5.5 years later, both tumors were pathologically and molecularly evaluated at the national central pathology review board and defined as ATRT in a syndromic patient, with strong evidence of a clonal origin of the two lesions. The patient was then treated according to SIOP guidelines and is now alive without evidence of disease 24 months after the detection of metastatic disease and 90 months after the original diagnosis.

**Conclusion:**

The report underscores the current utility of multiple comprehensive approaches for the correct diagnosis and clinical management of patients affected by rare and atypical brain neoplasms. Successful local control of disease and achievement of long-term survival is possible in ATRT patients even in the setting of rhabdoid tumor predisposition syndrome, infant age at diagnosis and metastatic spread of disease, thus justifying the efforts for the management of this severe condition.

## Background

*SMARCB1/INI1* is a highly conserved core subunit of the SWI/SNF family of ATP-dependent chromatin remodeling complexes [1] and acts as a tumor suppressor gene inactivated in malignant rhabdoid tumors (MRT) of childhood, encompassing renal, soft-tissue and brain cancers [2] and characterized by *SMARCB1/INI1* genetic inactivation as a primary, recurrent event [3,4]. Notably, a consistent fraction of cases carries de-novo *SMARCB1/INI1* constitutional mutations in the setting of the so called “rhabdoid tumor predisposition syndrome” [5,6].

MRTs of the brain, known as Atypical Teratoid Rhabdoid Tumor (ATRT), were initially considered as a medulloblastoma subgroup with poor prognosis and have been suggested as a separate entity since 1996 [7,8], but only recently systematic *SMARCB1/INI1* immunohistochemistry and mutational screening in newly diagnosed pediatric brain tumors [9-11] has allowed the definition of the incidence, clinical, pathologic and molecular features of ATRT [11]. ATRT represents an aggressive neoplasm of childhood with a dismal prognosis, presenting a median overall survival of less than 12 months and less than 20% progression-free survival at 1 year from diagnosis. Particularly, infant age [12,13], metastasis at diagnosis [12] and the status of carrier of rhabdoid tumor predisposition syndrome [5,14,15] invariably lead ATRT patients to rapid lethal outcome, with less than 30% overall survival at 1 year. To our knowledge, no long-term survival patient has been reported so far presenting all these three adverse prognosis’ features.

## Case presentation

### Patient’s history and clinical features

**Figure 1:**
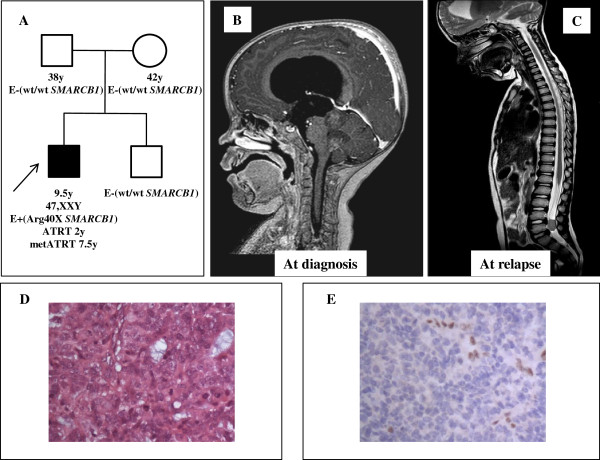
***A*, Schematic representation of the family tree of index case.** Karyotype and *SMARCB1/INI1* molecular evaluation (**E**) is reported (wt= wild type; Arg40X denotes the constitutional mutation found in index case). Present age and age of onset of clinical symptoms is given in years (y). ***B***, Brain MRI of primary ATRT lesion. ***C***, MRI of metastatic lesion. ***D***, Hematoxylin eosin staining and ***E***, *SMARCB1/INI1* staining of ATRT tumor from index case, showing absence of *SMARCB1/INI1* protein expression in cancer cells.

**Figure 2:**
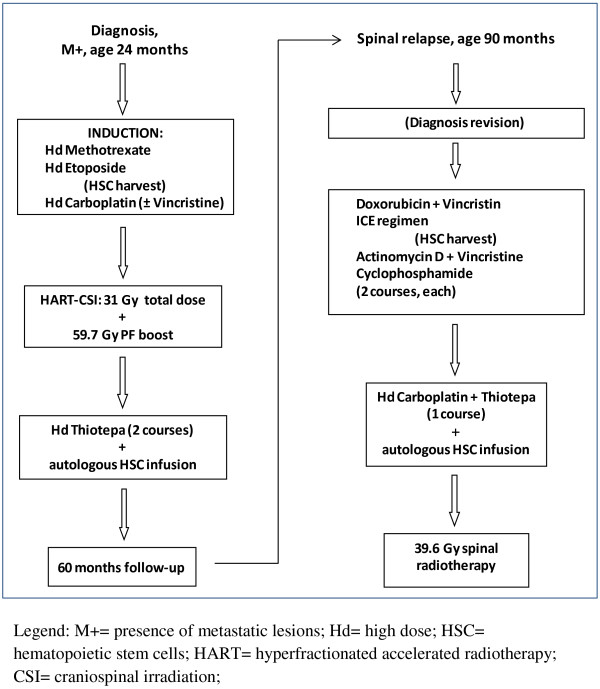
Schedules of chemotherapy and radiotherapy.

The patient (Figure 1A), a 24 months-old boy affected by mosaic Klinefelter syndrome (47, XXY [14]/46,XY [65]) presented with cerebellar and endocranial hypertension symptoms. Initial MRI examination showed a 3×4 cm vermian nodule in the axial plane influencing tri-ventricle hydrocephalon and transependimal liquoral re-absorpion (Figure 1B). He was submitted to surgery with the intent of complete tumor removal but post-operative staging showed multiple hemispherical cerebellar nodules, concurrent spinal metastases at the lumbar and caudal tracts, and cerebrospinal fluid dissemination. Final histological diagnosis, performed at the original neuropathology unit, was medulloblastoma. He was sent to our Unit for adjuvant treatment that, according to the Italian Association of Hematology and Oncology (AIEOP) protocol for high-risk infant medulloblastoma [16,17] (Figure 2), consisted of sequential high-dose (hd) methotrexate and vincristine, hd-etoposide, hd-cycloposphamide and hd-carboplatin delivered within a 2 month time without obtaining a satisfying metastatic tumor response. Craniospinal irradiation according to the hyperfractionated accelerated radiotherapy schedule [16] was therefore delivered, with a total dose of 31.2 Gy to the neuraxis and a boost on posterior fossa up to a total dose of 59.7 Gy. Complete response was eventually obtained and two subsequent consolidation courses with high-dose thiotepa were delivered thereafter, followed by rescue autologous hemopoietic stem cells that were harvested after hd-etoposide in the pre-radiant phase.

The child was thereafter asymptomatic until November 2010 when he complained of gait disturbances and lower back pain. The MRI performed showed an intrarachideal spinal lesion extended from L3 to L5 (Figure 1C) that was surgically excised sub-totally. The staging was repeated with whole CNS MRI and cytological cerebrospinal fluid examination and did not show other neoplastic sites. Analysis of this second tumor under central pathology review board concluded for the diagnosis of ATRT, supported by negative *SMARCB1/INI1* staining. Central pathology re-evaluation of primary tumor revealed negative *SMARCB1/INI1* staining, prompting a reassessment of the original diagnosis to ATRT (Figure 1D-E). Parents accepted the new treatment (Table 1), proposed based on International Society of Pediatric Oncology (SIOP) guidelines for rhabdoid tumors, consisting of chemotherapy alternating adriamycin and vincristine, carboplatin plus etoposide and ifosfamide (ICE regimen) and actinomycin D, cyclophosphamide and vincristine, leading to complete response of spinal residual disease. Due to previous craniospinal irradiation, no intratechal chemotherapy was planned. The systemic treatment finally included one myeloablative course with high-dose carboplatin and thiotepa. Following restoration from aplastic period he was re-irradiated on the spinal tumor bed. Radiation was delivered to the spine and conus medullaris from L3 to S4 and reached a total dose of 39.6 gray (Gy) with a standard fractionation of 1.8 Gy/day. The child is now 114 months old and is alive without evidence of disease 24 months after spinal relapse and 90 months after the original diagnosis.

**Table 1:** Microsatellite alleles and sequence polimorphisms of mitochondrial DNA hypervariable regions (HV1 and HV2) detected in constitutional DNA, primary tumor and metastasis of ATRT index case

**Marker**	**Costituzional DNA**	**Primary tumor**	**Metastatic tumor**	**Mb position (chr22 start)**
**D22S427**				
***(22q11.21)***	97 - 97	97	97	18,591,317
**D22S257**				
***(22q11.23)***	120 - 128	*128*	*128*	23,568,429
**D22S1174**				
***(22q11.23)***	139 - 141	*139*	*139*	24,488,486
**D22S1154**				
***(22q12.1)***	264 - 266	*264*	*264*	26,617,527
**D22S1163**				
***(22q12.1)***	148 - 162	*162*	*162*	27,918,651
**HV1**	-	16183C	16183C	-
		16189C	16189C	
**HV2**	-	263 G	263 G	-
		309.3C	309.3C	
		315.1C	315.1C	

The patient is at present in the full-course of the primary school and is assisted by a tutor. Indeed, the cognitive and developmental status of the patient was influenced by Klinefelter syndrome, whose symptoms were not properly and early addressed due to cancer occurrence. During the course of the ATRT disease, the patient suffered hydrocephalus at diagnosis and he was affected by posterior fossa syndrome in the post-operative period. He had a direct neuro-cognitive assessment 2 years after the end of the first treatment and displayed a full-scale IQ of 68, verbal IQ of 72 and performance IQ of 72, but we lack basal evaluations. For all these reasons, it is not possible to draw any conclusion on the morbidity and cognitive/developmental impact of specific therapies applied.

### Patient’s molecular features

**Figure 3:**
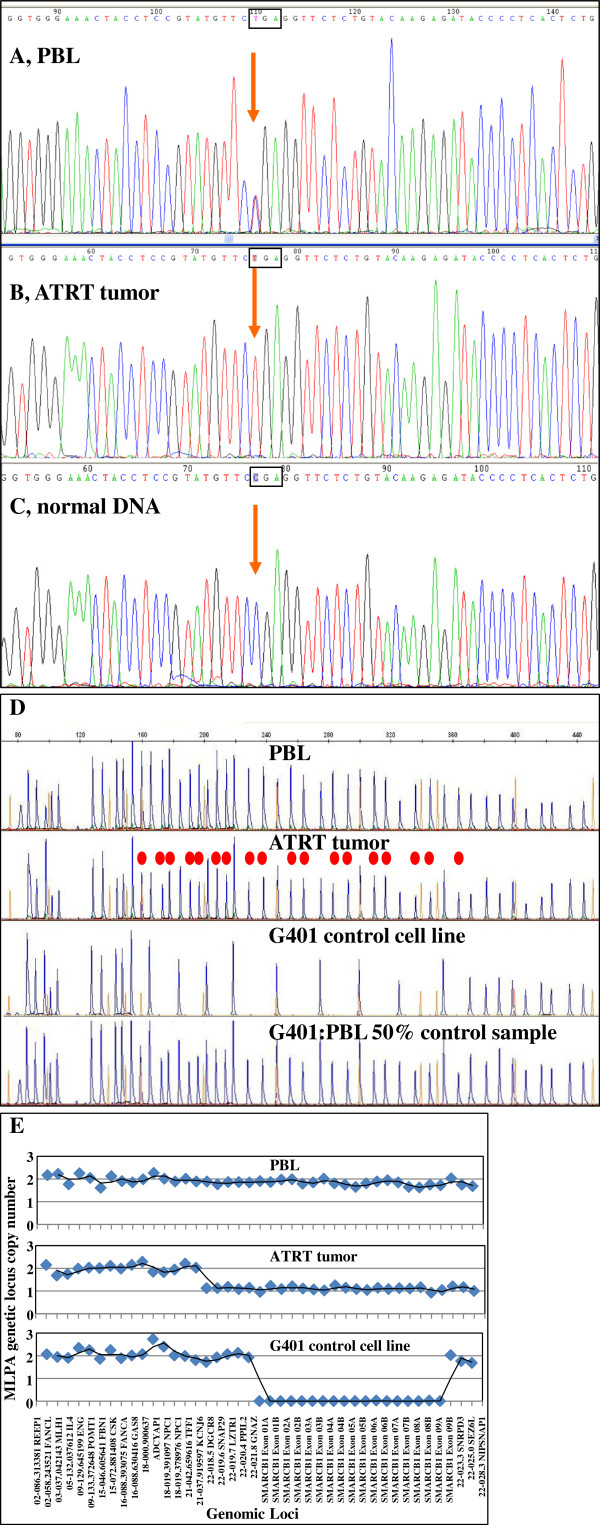
***A-C*, Electropherogram of normal and tumor DNA-derived sequences showing the presence of heterozygous exon 2 c.**118C>T (Arg40X) in (***A***) patient’s constitutional DNA and the same homozygous mutation **(*B*)** in tumor samples. ***C*,** Parental sample, displaying normal DNA sequence. ***D-E***, Fragment analysis (***D***) and histogram (***E***) of MLPA results for *SMARCB1/INI1* gene dosage analysis in primary tumor tissue and in control samples. Red dots indicate peaks corresponding to SMARCB1/INI1 fragments.

Negative immunostaining for *SMARCB1/INI1* protein prompted us to further investigate *SMARCB1/INI1* gene status in Carnoy’s fixed (primary tumor) and formalin-fixed (spinal metastasis), paraffin-embedded biopsies, in order to corroborate the pathological results. *SMARCB1/INI1* exon amplification and sequencing revealed the presence of a homozygous exon 2 c.118C>T (Arg40X) mutation in both the primary and metastatic tumor lesions (Figure 3A).

Investigation of patient’s peripheral blood-derived DNA revealed the presence of the same mutation in heterozygosity (Figure 3B), thus establishing the constitutional origin of the mutation. Both parents and the younger brother carried instead a wild-type sequence (Figure 1C), suggesting the de-novo origin of the mutation in the index case. The c.118C>T (Arg40X) mutation has already been described in malignant rhabdoid tumor [18] and leads to the creation of a premature stop codon in *SMARCB1/INI1* exon 2.

Loss of the second allele in tumor tissue as a second hit for *SMARCB1/INI1* inactivation was suggested by sequencing results (Figure 3A) and further supported by multiplex ligation-dependent probe amplification (MLPA) gene dosage analysis that revealed hemizygous deletion of the entire *SMARCB1/INI1* gene and flanking chromosome 22q probes in both tumors (Figure 3D-E). Normal DNAs and samples carrying known *SMARCB1/INI1* imbalances were used as controls. The presence of *SMARCB1/INI1* hemyzigous deletion was confirmed also by Real-time quantitative PCR analysis of *SMARCB1/INI1* copy number by means of relative quantification using standard curve method (not shown).

In order to study the relationship between the two tumor lesions, they were further investigated by analyzing the status of 22q microsatellite loci and mitochondrial DNA hypervariable regions. The results (Table 1) indicate a complete identity of the two lesions and therefore support a clonal origin and strongly suggest that the spinal tumor represents a metastatic spread of the primary tumor.

## Conclusions

Malignant rhabdoid tumors are lethal neoplasms of infancy, which can affect renal and extrarenal locations, including soft tissues and brain [19,20]. Notably, these tumors also represent the sole manifestation of a heritable cancer predisposition syndrome caused by constitutional alterations of *SMARCB1/INI1* tumor suppressor gene [5,6]. Overall survival in rhabdoid tumor patients is dismal and survival is worst in the setting of infant patients and patients affected by rhabdoid tumor predisposition syndrome [5]. In malignant rhabdoid tumors affecting the brain (called Atypical Teratoid Rhabdoid Tumors – ATRT), similar results are reported and germline mutations are associated with fatal outcome within two-years from diagnosis [14].

Outcome improvements in ATRT have been reported with the adoption of high-dose, multimodality chemotherapy regimens [13,21-23]. Indeed, case reports of long-term survival in ATRT have been described [24-26] but, to our knowledge, no case reported so far carried constitutional *SMARCB1/INI1* alterations as our infant index case.

We here describe a long-term surviving patient affected by syndromic ATRT and who is alive 7.5 years after the original diagnosis and 2 years after onset of a spinal metastatic lesion. All the analyses performed showed an identical genetic profile between the primary and metastatic ATRT lesions. Although we cannot formally exclude that the two lesions represent the occurrence of two independent primary ATRTs, our genetic analyses as well as the clinical history of the patient, characterized by the presence of metastatic spread since the occurrence of the primary tumor, make this hypothesis very unlikely and support a clonal origin of the two lesions.

ATRT tumor from proband case displayed a biallelic inactivation of SMARCB1/INI1 gene by heterozygous loss of one allele and Arg40X mutation in the exon 2 of the second allele. This mutation, by causing a premature stop codon, is of obvious pathogenic consequence and has already been reported in the spectrum of mutations occurring in malignant rhabdoid tumors [18]. Although to our knowledge this is the first report of Arg40X occurrence in a syndromic ATRT patient, such mutation has been already reported in syndromic extracerebral MRT and has functional consequences overlapping those of other exon 2 mutations previously reported in syndromic ATRT patients, such as Arg53X [5]. These data indicate that Arg40X mutation can occur in both sporadic and syndromic, and in both cerebral and extracerebral MRTs, thus suggesting that no specific genotype-phenotype correlation exists.

The patient under study was affected by mosaicism for Klinefelter syndrome, the most common human sex chromosome disorder, presenting 18% of peripheral blood lymphocytes with chromosome X aneuploidy and this report represents, to our knowledge, the first association of ATRT with Klinefelter. Despite individual case reports emphasized the association between Klinefelter and specific cancers’ risk, reviews of epidemiological data do not support a generalized increased risk of cancer in Klinefelter patients [27,28], which remains significant only for breast cancer if compared to the general population of males but not of females [27]. In addition, there is no evidence of recurrent X chromosome abnormalities or X-chromosome gene(s) mutation in ATRT tumor samples analyzed by whole-genome approaches [4,29], so we suggest that the occurrence of ATRT in our patient is unrelated to Klinefelter syndrome. Furthermore, in cancer patients Klinefelter is associated with increased risk of cancer mortality [27] and, although the risk is variable in different cancer types, Klinefelter did not display a protective effect in any individual tumor type investigated. Therefore, we consider very unlikely the possibility that the constitutional karyotype of the patient may have positively affected the course of ATRT, being responsible for the observed long-term survival.

The patient presents cognitive and developmental delay, as a consequence of Klinefelter syndrome as well as ATRT treatment complications. In this complex scenario, the lack of multiple neuropsychological assessments during the different disease stages impedes to draw any conclusion on the morbidity and cognitive/developmental impact of specific therapies applied.

To conclude, the observed successful local control of disease and achievement of long-term survival in our molecularly-proven ATRT patient even in the setting of rhabdoid tumor predisposition syndrome justifies the efforts to advance the management of this severe condition.

## Materials and methods

### Exon PCR and sequencing

Mutational analysis was performed by exon amplification and sequencing as previously described [30]. Primers and PCR conditions are available upon request. PCR products were enzymatically purified with ExoSap (Affymetrix) and sequenced with BigDye Terminator chemistry (Applera). Sequencing products were run on an ABI3130xl genetic analyzer (Applera) and electropherograms were visually inspected. The two strands of mitochondrial DNA hypervariable regions HVS-I and HVSII were amplified and sequenced following standard procedures [31].

#### Multiplex ligation-dependent probe amplification

For Multiplex ligation-dependent probe amplification (MLPA), tumor DNA (100 ng) was subjected to DNA copy number analysis using MLPA kits P258-B1, (MRC-Holland), following manufacturer instructions, together with normal DNA samples and cancer cell line samples with known *SMARCB1/INI1* gene copy number alterations as controls. Fragment separation was performed on an ABI3130xl genetic analyzer (Applera). Raw data peak pattern evaluation was performed using GeneMapper software (Applera) and Coffalyser software was used for data analysis (MRC-Holland). Normal DNAs were used as calibrators and samples with known *SMARCB1/INI1* copy number alterations were used as controls for the sensitivity of the test.

#### Real-time quantitative PCR

Analysis of *SMARCB1/INI1* copy number by real-time quantitative PCR was performed by means of relative quantification using standard curve method [32], using TaqMan assays 4401631 (Applera) for RPPH1 endogenous control gene and Hs01497967_cn (Applera) for *SMARCB1/INI1*. Standard curve was constructed using normal control DNA and 22q normal copy FFPE controls served as calibrator.

#### Microsatellites

Microsatellite alleles’ analysis was performed by capillary electrophoresis of fluoresceinated amplification products obtained from peripheral blood- and tumor-derived DNA. PCR products were run on an ABI3130xl genetic analyzer (Applera) and raw data were acquired with GeneMapper software and the peak pattern was visually evaluated.

### Consent

Parents have given consent for the case report to be published.

## Competing interests

The authors declare that they have no competing interests.

## Authors’ contributions

PM, MB, LG, RM carried out the molecular studies. IS, VB, LG, MM carried out the clinical management of the patient. AMB, BP, FG carried out pathologic assessments. All authors read and approved the final manuscript.

## Pre-publication history

The pre-publication history for this paper can be accessed here:

http://www.biomedcentral.com/1471-2407/13/100/prepub
